# Supplementary material for: Structural Basis of Peptide-Based Antimicrobial Inhibition of a Resistance-Nodulation-Cell Division Multidrug Efflux Pump
Source: Microbiol Spectr. 2022 Sep 19;10(5):e02990-22. doi: 10.1128/spectrum.02990-22 (PMC9603588; doi:10.1128/spectrum.02990-22)
Supplement: Supplemental file 1 — Supplemental material. Download spectrum.02990-22-s0001.pdf, PDF file, 1.6 MB [file spectrum.02990-22-s0001.pdf]

Supplemental Material

Structural Basis of Peptide-Based Antimicrobial Inhibition of a Resistance-Nodulation-Cell Division Multidrug Efflux Pump

Meinan Lyu[1], Julio C. Ayala[2], Isabella Chirakos[1], Chih-Chia Su[1], William M. Shafer[2,3] and Edward W. Yu[1,*]

[1]Department of Pharmacology, Case Western Reserve University School of Medicine, Cleveland, OH 44106, USA.

[2]Department of Microbiology and Immunology and the Emory Antibiotic Resistance Center, Emory University School of Medicine, Atlanta, Georgia 30322.

[3]Laboratories of Microbial Pathogenesis, VA Medical Center, Decatur, Georgia 30033.

[*]To whom correspondence should be addressed. Email: edward.w.yu@case.edu

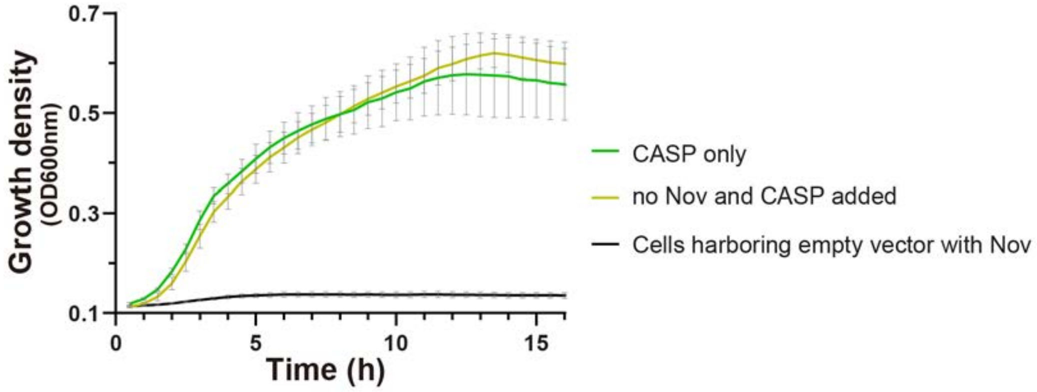

Figure S1. Growth of *E. coli* BL21(DE3)Δ*acrB*/pACYCΩ*mtrCDE* cells. This figure indicates the growth of cells in the absence of both Nov and CASP (splitpea curve), and in the presence of 2.0 mg/ml CASP only (green curve). The growth of *E. coli* BL21(DE3)Δ*acrB*/pACYC cells, which harbor the empty vector, in the presence of 75 μg/ml Nov is shown as a black curve.

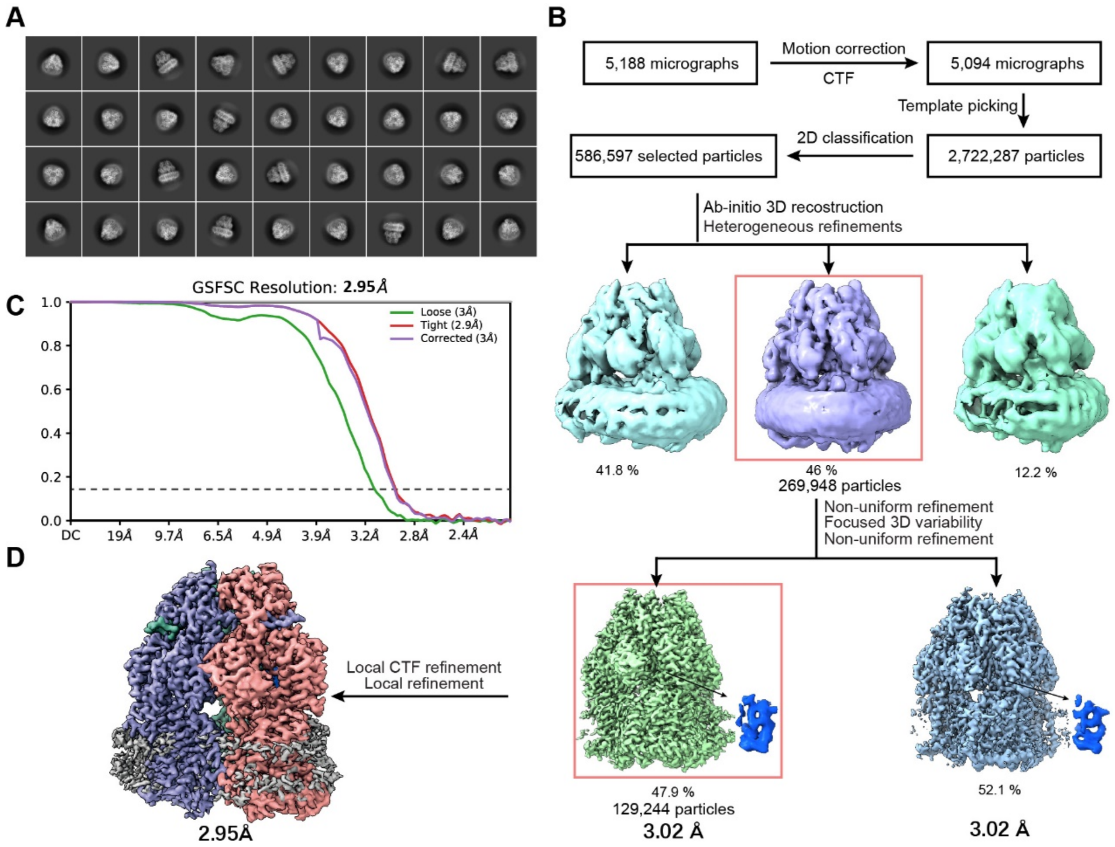

Figure S2. Cryo-EM structure of the $\mathrm{MtrD_{CR103}}$ efflux pump in complex with CASP. (A) Representative 2D classes. (B) Data processing flowchart with particle distributions. A red box indicates the class used for further refinement. (C) Fourier shell correlation (FSC) curves showing a resolution of 2.95 Å. (D) Cryo-EM density maps of CASP bound $\mathrm{MtrD_{CR103}}$. The “access”, “binding” and “extrusion” protomers of the $\mathrm{MtrD_{CR103}}$ are colored slate, salmon and green, respectively. Densities contributed to the nanodisc belt are colored gray. Densities of the CASP peptide found in the “binding” protomer of $\mathrm{MtrD_{CR103}}$ are in blue color.

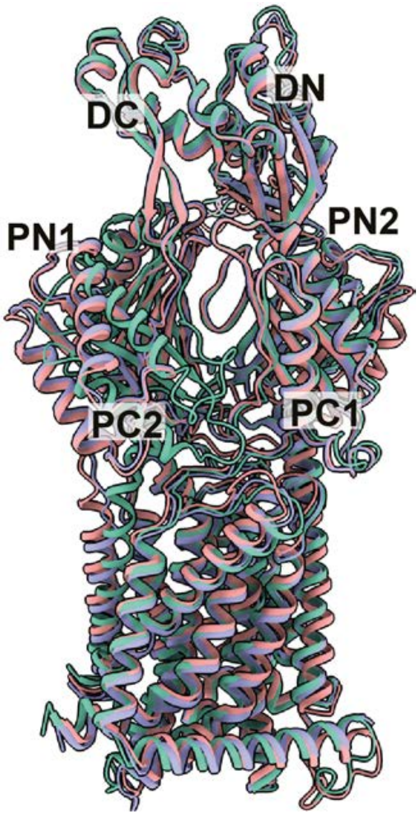

Figure S3. Superimposition of the “binding”, “extrusion” and “access” protomers of MtrD $_{CR103}$. Pairwise superimpositions of protomers (for 1,039 Cα atoms) of “access” and “binding”, “access” and “extrusion”, and “binding” and “extrusion” gave rise to r.m.s.d. values of 2.03, 3.18 and 3.42, respectively.

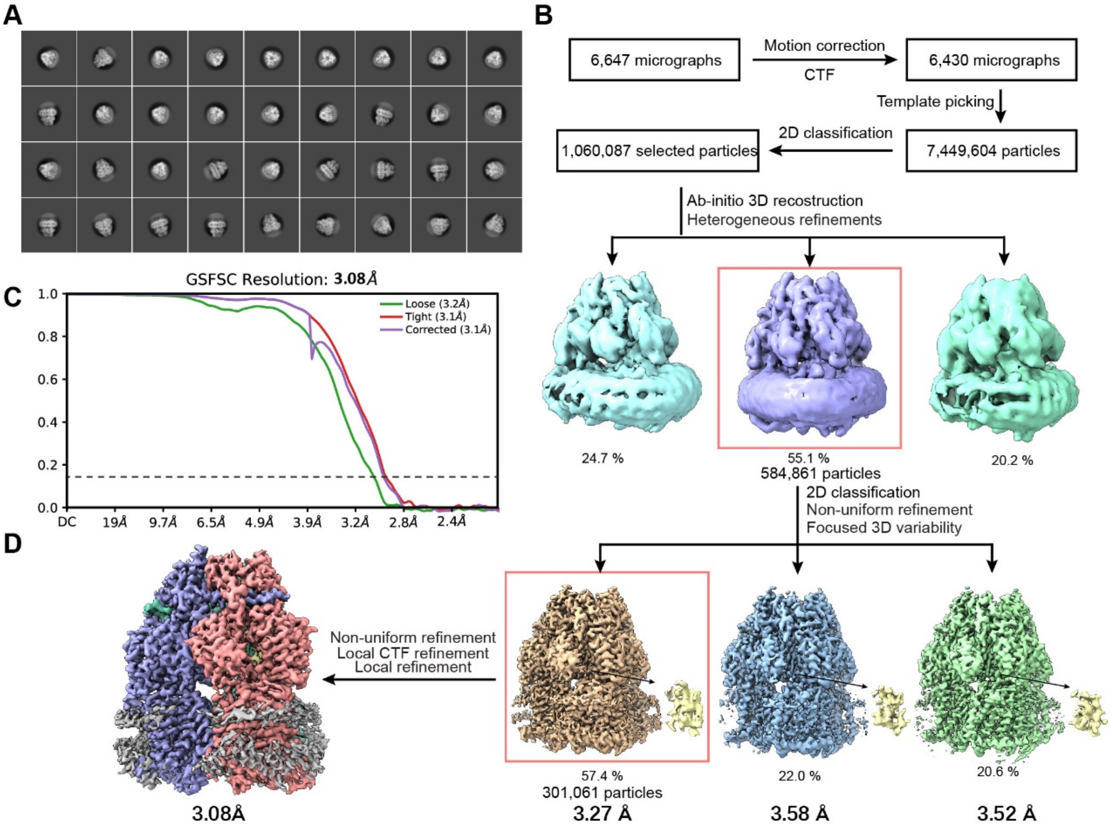

Figure S4. Cryo-EM structure of the $MtrD_{CR103}$ efflux pump in complex with Col. (A) Representative 2D classes. (B) Data processing flowchart with particle distributions. A red box indicates the class used for further refinement. (C) Fourier shell correlation (FSC) curves showing a resolution of 3.08 Å. (D) Cryo-EM density maps of Col bound $MtrD_{CR103}$. The "access", "binding" and "extrusion" protomers of the $MtrD_{CR103}$ are colored slate, salmon and green, respectively. Densities contributed to the nanodisc belt are colored gray. Densities of the Col peptide found in the "binding" protomer of $MtrD_{CR103}$ are in yellow color.

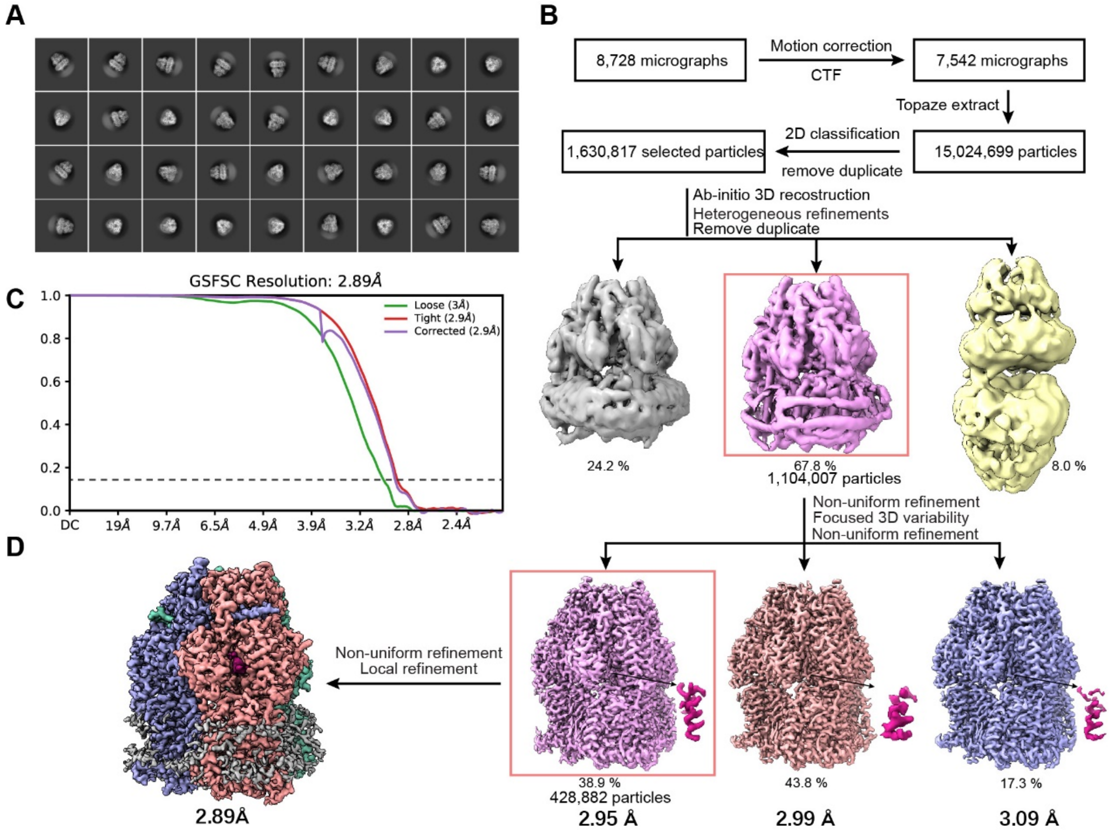

Figure S5. Cryo-EM structure of the $MtrD_{CR103}$ efflux pump in complex with LL. (A) Representative 2D classes. (B) Data processing flowchart with particle distributions. A red box indicates the class used for further refinement. (C) Fourier shell correlation (FSC) curves showing a resolution of 2.89 Å. (D) Cryo-EM density maps of LL bound $MtrD_{CR103}$. The "access", "binding" and "extrusion" protomers of the $MtrD_{CR103}$ are colored slate, salmon and green, respectively. Densities contributed to the nanodisc belt are colored gray. Densities of the LL peptide found in the "binding" protomer of $MtrD_{CR103}$ are in magenta color.

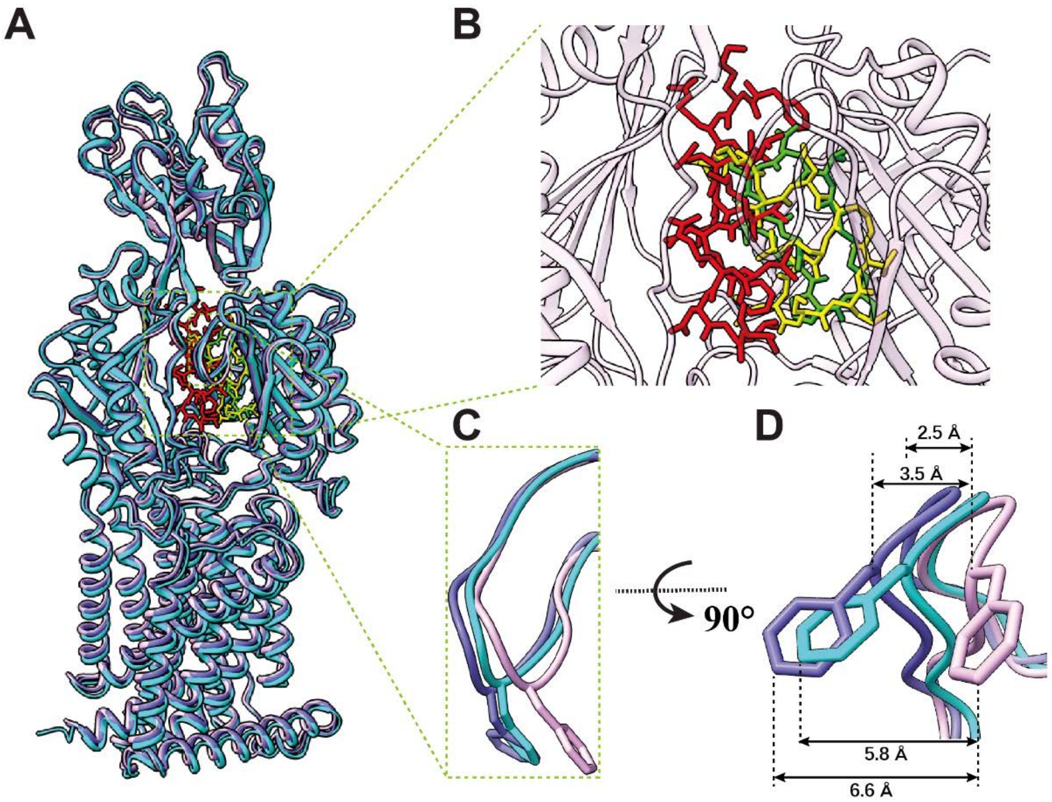

Figure S6. Comparison of the CASP, Col and LL binding sites. (A) Superimposition of the "binding" protomers of $MtrD_{CR103}$-CASP (slate), $MtrD_{CR103}$-Col (cyan) and $MtrD_{CR103}$-LL (pink). (B) The peptide binding site. The bound CASP, Col and LL peptides are colored green, yellow and red, respectively. The secondary structural elements of the "binding" protomer of $MtrD_{CR103}$ are color pink. (C and D) Superimposition of the G-loop of $MtrD_{CR103}$-CASP (slate), $MtrD_{CR103}$-Col (cyan) and $MtrD_{CR103}$-LL (pink). This figure indicates that there is a major change in conformation of the G-loop in order to accommodate for the binding of these three different peptides.

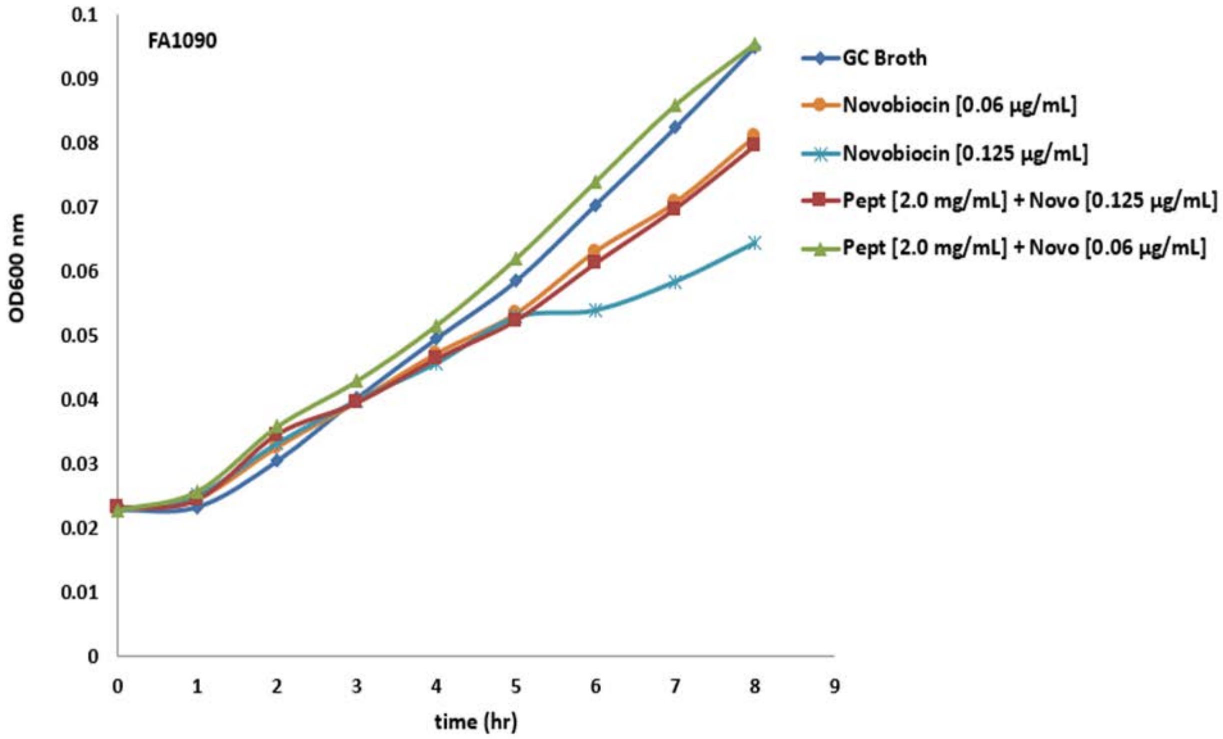

Figure S7. CASP does not sensitize *N. gonorrhoeae* to sub-lethal levels of Nov. 10^7 CFUs of gonococcal strain FA1090 were seeded in 100 µL of GC broth alone or containing CASP 2.0 mg/ml, novobiocin at sub-lethal levels (0.06 or 0.125 µg/ml) or a combination of CASP and novobiovin at indicated concentrations. Cells were incubated at 37°C, and growth was followed every hour by reading OD_{600} on a plate reader.

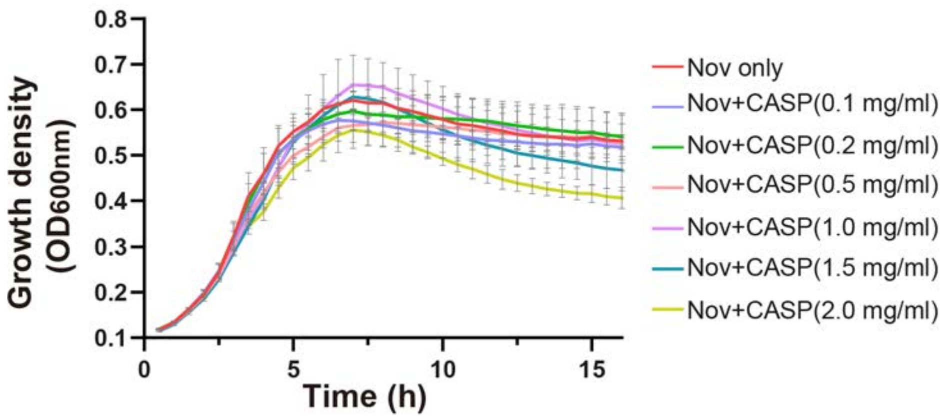

Figure S8. Growth of *N. subflava* ATCC 49275 cells in the presence of 0.5 µg/ml Nov. The MIC of Nov for *N. subflava* ATCC 49275 is 1 µg/ml. The growth curves of cells supplemented with 0, 0.1, 0.2, 0.5, 1.0, 1.5 and 2 mg/ml CASP are colored red, slate, green, wheat, violet, blue and splitpea, respectively.

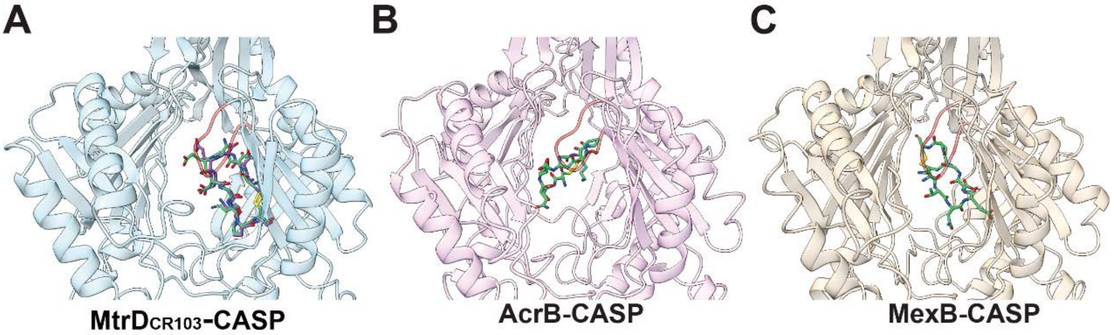

Figure S9. *In silico* docking of CASP to the "binding" protomer of $MtrD_{CR103}$, AcrB and MexB. (A) Predicted CASP binding site for *N. gonorrhoeae* $MtrD_{CR103}$ based on the cryo-EM structure of the "binding" protomer of $MtrD_{CR103}$ from this study. The predicted CASP binding site is in excellent agreement with that from the cryo-EM structure of the $MtrD_{CR103}$-CASP complex. The bound CASP molecule based on AutoDock Vina software and the cryo-EM structure are in green and purple sticks, respectively. The G-loop is colored pink. The calculated binding affinity of CASP is -9.4 kcal/mol. (B) Predicted CASP binding site for *E. coli* AcrB based on the X-ray structure of the "binding" protomer of AcrB (PDB ID: 2DRD). The bound CASP molecule based on AutoDock Vina is in green sticks. The G-loop is colored pink. The calculated binding affinity of CASP is -8.5 kcal/mol. (C) Predicted CASP binding site for *P. aeruginosa* MexB based on the X-ray structure of the "binding" protomer of MexB (PDB ID: 2V50). The bound CASP molecule based on AutoDock Vina is in green sticks. The G-loop is colored pink. The calculated binding affinity of CASP is -6.6 kcal/mol.

Table S1. Anti-MtrD peptides identified by phage display.

Name	Anti-MtrD Peptide sequence
ADP1	C N W M I N K E C
ADP2	C N A G H L S Q C
ADP3	C N M H T P M V C
ADP4	C S G L T R P T C
ADP5	C I V P S S K S C
ADP6	C P K G D E N T C

Table S2. Loss of MtrD enhances gonococcal susceptibility to Nov.

Strain	MIC (µg/ml)
FA19	0.25
FA19 *mtrD::kan*	0.06
WHO X	1.0
WHO X *mtrD::kan*	0.25

Table S3. Cryo-EM data collection, processing and refinement statistics.

Data set	$MtrD_{CR103}$-CASP	$MtrD_{CR103}$-Col	$MtrD_{CR103}$-LL
**Data collection and processing**			
Magnification	81,000	81,000	81,000
Voltage (kV)	300	300	300
Electron Microscope	Krios-GIF-K3	Krios-GIF-K3	Krios-GIF-K3
Defocus range (μm)	-0.75 to -1.75	-1.75 to -2.5	-1.0 to -2.25
Total exposure time (s)	2.6	2.8	2.6
Pixel size (Å)	1.08	1.12	1.08
Total dose (e^-/Å^2)	40	40	40
Number of frames	40	40	40
Dose rate (e^-/phys. Pixel/s)	18.0	17.98	18.035
No. of initial micrographs	5,188	6,647	8,728
No. of initial particles	2,722,287	7,449,604	15,024,699
No. of final particles	129,244	301,061	428,882
Symmetry	C1	C1	C1
GSFSC Resolution (Å)	2.95	3.08	2.89
FSC threshold (0.143)			
Density modification resolution	2.35	2.48	2.29
**Refinement**			
Model resolution cut-off (Å)	2.35	2.48	2.29
Model composition			
No. of Protein residues	3130	3122	3172
No. ligands	27	24	23
RMSD[a]			
Bond lengths (Å)	0.005	0.003	0.003
Bond angles (°)	0.982	0.493	0.535
**Validation**			
Mol Probity score	2.66	2.05	1.55
Ramachandran plot (%)			
Favored (%)	94.39	95.70	96.78
Allowed (%)	5.51	4.30	3.19
Disallowed (%)	0	0	0
CC[b] Mask	0.84	0.85	0.82

[a]root mean square deviation

[b]correlation coefficient
